# Enhanced Soluble Expression of Linoleic Acid Isomerase by Coordinated Regulation of Promoter and Fusion Tag in *Escherichia coli*

**DOI:** 10.3390/foods11101515

**Published:** 2022-05-23

**Authors:** Baixi Zhang, Tong Zhu, Xintian Huang

**Affiliations:** 1 School of Food Science and Technology, Jiangnan University, 1800 Lihu Road, Wuxi 214122, China; 15388545375@163.com (T.Z.); 18637379637@163.com (X.H.); 2 National Engineering Research Center for Functional Food, Jiangnan University, 1800 Lihu Road, Wuxi 214122, China

**Keywords:** PAI, promoter, fusion tag, soluble expression, enzyme activity

## Abstract

PAI is a linoleic acid isomerase from *Propionibacterium acnes* and is the key enzyme in the synthesis of *trans*10, *cis*12-conjugated linoleic acid. However, the majority of the expressed PAI in *Escherichia coli* occurs in its nonfunctional form in inclusion bodies, limiting the biosynthesis of conjugated linoleic acid. In an attempt to improve the solubility of recombinant PAI in *Escherichia coli*, three promoters representing different transcriptional strengths (T7, CspA, and Trc), paired with three fusion tags, (His6, MBP, and Fh8), respectively, were investigated in this study. Among the nine recombinant strains, *Escherichia coli* BL21 (DE3) (pET24a-Mpai), containing the T7 promoter and MBP fusion tag, led to a considerable increase in PAI solubility to 86.2%. MBP-PAI was purified 41-fold using affinity column chromatography. The optimum catalytical conditions of MBP-PAI were 37 °C and pH 7.5 with the addition of 1 mmol/L Tween-20. Most of the tested metal ions inhibited MBP-PAI activity. The apparent kinetic parameters (*K*m and *V*max) were measured with linoleic acid concentrations ranging from 71 μM to 1428 μM. The substrate linoleic acid did not exert any inhibitory effect on MBP-PAI. The *K*m of MBP-PAI was 253.9 μmol/L, and the *V*max was 2253 nmol/min/mg. This study provided a new method for improving the solubility of the recombinant linoleic acid isomerase in *Escherichia coli*.

## 1. Introduction

Conjugated linoleic acid (CLA) is a group of polyunsaturated fatty acids that is widely used in foods and dietary supplements. CLA has different structural isomers. *Trans*-10, *cis*12-conjugated linoleic acid (*t*10, *c*12-CLA) is an octadecadienoic acid with two conjugated double bonds that are found naturally in ruminant animal food products [1]. It has been applied in food products and the medical industry for its physiological functions, including antiobesity, antiatherosclerosis, improvement of diabetes, and anticancer [2,3,4,5,6,7].

**Figure 1:**
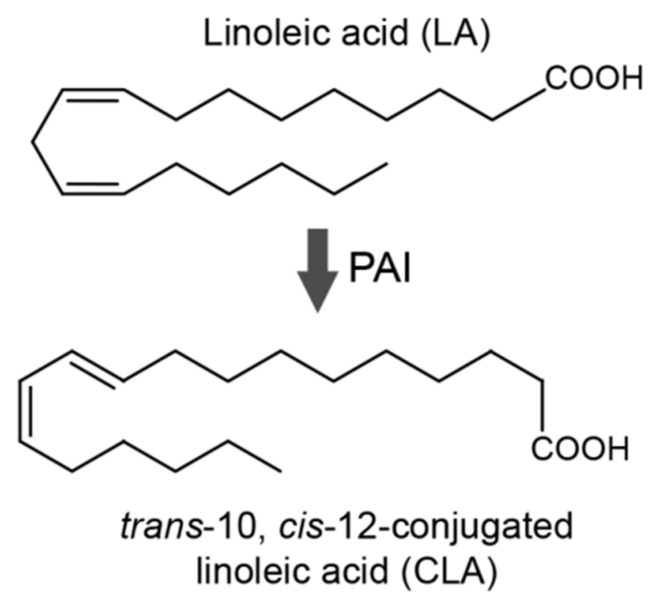
Conversion from linoleic acid to *trans*-10, *cis*-12-conjugated linoleic acid by PAI, a linoleic acid isomerase from *Propionibacterium acnes* (*P. acnes*).

Biosynthesis is a potential manufacturing method for CLA production and has gained growing attention. PAI, a linoleic acid (LA) isomerase from *Propionibacterium acnes* (*P. acnes*), is the only enzyme known to catalyzes the conversion of LA into *t*10, *c*12-CLA (Figure 1) [8]. However, *P. acnes* is a pathogenic microorganism unsuitable for CLA production. Therefore, various microorganisms were investigated in an attempt to increase the PAI yield. Still, the recombinant PAI either showed low expression levels or low solubility (such as *Escherichia coli*, *Saccharomyces cerevisiae*, *Pichia pastoris*, *Yarrowia lipolytica*, and *Mortieralla alpina*) [9,10,11,12,13]. *Escherichia coli (E. coli)* remains the primary microorganism used in laboratory investigations and initial development for commercial activities. However, some recombinant proteins were produced as inclusion bodies due to the fast-folding kinetics in *E. coli* cells [14]. PAI expression in *E. coli* appears to follow a similar trend [10]. Studies have shown that the solubility of foreign proteins in *E. coli* was closely related to the promoter strength and the fusion partner compatibility [14,15,16]. Although coexpression with tags can effectively reduce the formation of the inclusion bodies, there is no universal fusion tag that can be applied to all proteins. Therefore, screening for a suitable promoter and fusion tag combinations may be an effective strategy to improve the soluble expression of PAI.

In this study, nine recombinant strains were constructed and assessed by pairing three promoters of varying transcriptional strengths (T7, CspA and Trc) and three fusion tags with both solubility and purification functions: MBP (maltose-binding protein), Fh8 (calcium-binding protein from Fasciola hepatica), and His6. The properties of the fusion protein with the highest solubility and activity were also investigated.

## 2. Materials and Methods

### 2.1. Materials

TransStart^®^ FastPfu DNA Polymerase was purchased from Transgen Biotech (Beijing, China). The restriction enzymes *Nde*I, *Xho*I, *Sac*I, *Hind*III, and *Xba*I were purchased from NEB (Ipswich, MA, USA). The pEASY-Uni Seamless Cloning and Assembly Kit and Plasmid miniprep kit were purchased from Transgen Biotech (Beijing, China). A gel extraction kit was obtained from Thermo Fisher Scientific (Shanghai, China). The MBPTrap^TM^ HP was purchased from GE Healthcare (Boston, MA, USA). Antibodies were synthesized by our team. The plasmids pCold IV, pET-24a(+), and pTrc99a were purchased from Takara (Dalian, China) and Novagen (Madison, WI, USA), respectively. The strains *E. coli* BL21 (DE3), *E. coli* BL21, and *E. coli* JM109 were purchased from NEB (Ipswich, MA, USA).

### 2.2. Construction of the Expression Vectors

**Figure 2:**
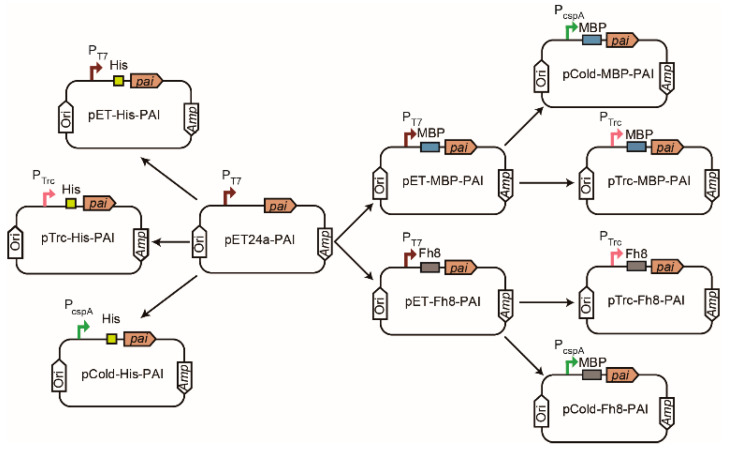
Construction of PAI expression vectors. PAI expression varied based on the promoters (T7, CspA, and Trc) and the fusion tags (MBP, His6, and Fh8).

The gene sequences of PAI (GenBank: AX062088), MBP-factorXa (GenBank: AHM36606.1), and Fh8-factorXa (GenBank: AF213970.1) were synthesized by Genscript Biotech Co. (Nanjing, China). The factor Xa recognition site encodes an amino acid sequence that can be identified and digested by the factor Xa protease. The synthesized PAI gene was inserted into the *Nde*I and *Xho*I restriction sites of the vector pET24a, resulting in the vector pET24a-PAI. Using pET24a-PAI as a template, the PAI gene with His6-tag sequences was amplified by polymerase chain reaction (PCR) using primers F1/R1 and F2/R2, respectively (Table 1). The PCR products were cloned into pET-24a(+) as a *Nde*I/*Xho*I fragment and into pCold IV and pTrc99a as a *Sac*I/*Hind*III fragment, resulting in the vectors pET-His-PAI, pCold-His-PAI, and pTrc-His-PAI. The vector pET-MBP-PAI was constructed by inserting the synthesized fragments of MBP-Factor Xa into the plasmid pET24a-PAI using the pEASY-Uni Seamless Cloning and Assembly Kit. Specifically, the plasmid pET24a-PAI was linearized by *Nde*I. The MBP-Factor Xa was amplified by PCR using primers F3 and R3 (Table 1), and using pUC57-(MBP-Factor Xa) as a template. Subsequently, the linearized vector and the PCR products were ligated based on the kit protocol. The vector pET-Fh8-PAI was constructed using primers F4 and R4 (Table 1) in a similar process to that for the pET-MBP-PAI. For the construction of the recombinant plasmids pCold-MBP-PAI and pTrc-MBP-PAI, the vectors pCold IV and pTrc99a were double-digested with *Hind*III and *Xba*I. Using pET-MBP-PAI as the template, the MBP-factorXa-PAI fragments were amplified with primers F5/R5 and F6/R6 (Table 1) and cloned into the linearized plasmids pCold IV and pTrc99a, respectively, using seamless cloning. Similarly, using pET-Fh8-PAI as the template, the Fh8-factorXa-PAI fragments were amplified with primers F7/R7 and F8/R8 (Table 1) and cloned into pCold IV and pTrc99a to generate the expression vectors pCold-Fh8-PAI and pTrc-Fh8-PAI. All the recombinant vectors used in this study are shown in Figure 2. Finally, the positive clones were selected through PCR and confirmed by DNA sequencing. The constructed pET-24a(+) series plasmids were transformed into *E. coli* BL21 (DE3), the pCold IV series plasmids were transformed into *E. coli* BL21, and the pTrc99a series plasmids were transformed into *E. coli* JM109 for expression.

### 2.3. Recombinant Protein Expression

A seed culture was prepared by inoculation with a single colony of recombinant *E. coli* strains and grown at 37 °C overnight in 5 mL of Luria–Bertani broth (1% peptone, 0.5% yeast extract, and 1% sodium chloride; pH 7.0). Ampicillin (100 μg/mL) was used for the pColdIV system and the pTrc99a system, while 50 μg/mL of kanamycin was used for the pET-24a(+) system. About 500μL of seed culture was subcultured into 50 mL of Luria–Bertani broth at 37 °C and 200 rpm until its OD600 reached 0.6. Isopropyl-β-D-thiogalactopyranoside (IPTG) was added to achieve a final concentration of 0.1 mM. The induction conditions depended on the expression vectors used (Table 2). For the pCold system, the shaker was immediately cooled down to 15 °C and maintained for 30 min before adding IPTG. The optimal induction conditions are displayed in Table 2.

### 2.4. SDS-PAGE Analysis of Recombinant Proteins

The recombinant cells were harvested by centrifugation at 10,000× *g* for 10 min at 4 °C. The cells were washed and suspended in a lysis buffer (100 mmol/L Tris-HCl, 10 mmol/L NaCl, 1 mM EDTA; pH 7.4) and were then disrupted by ultrasonication on ice. All of the soluble protein fraction was separated from the total protein by centrifugation at 14,000× *g* at 4 °C for 15 min, and the protein concentration was measured respectively using a BCA Protein Assay Kit (Beyotime, Shanghai, China). The total protein and the soluble protein fraction were analyzed using 12% sodium dodecyl sulfate-polyacrylamide gel electrophoresis (SDS-PAGE). The band intensity volumes were observed to assess protein expression. The quantity of recombinant protein was determined (as a percentage of total *E. coli* protein) by analysis of Coomassie-Blue-stained SDS polyacylamide gels using the image-analysis software Quantity One (BioRad, Hercules, CA, USA).

### 2.5. Measurement of Total PAI Activity with Cell Lysate

The total PAI activity with cell lysate was represented by the conversion ratio of LA into CLA. The reaction system consisted of 100 µL of recombinant cell lysate, 900 µL of buffer F (100 mmol/L Tris-HCl, 10 mmol/L NaCl, 1 mM EDTA; pH 7.4), and 10 µL of 20 µg/µL LA in a shaker at 37 °C for 1 h. The complex composition of the cell lysate had a negative impact on the measurement of CLA content. Therefore, gas chromatography was applied to measure the total PAI activity with cell lysate. For fatty acid methylation, 400 µL of methanol and 20 µL of (trimethylsilyl)-diazomethane was added to the samples. After 15 min of incubation at room temperature, 5 µL of 5% acetic acid was added to inactivate the diazomethane. The samples were dried, and the FA methyl esters were dissolved in 1 mL of N-hexane and analyzed using a GC-2010 (Shimadzu Co., Kyoto, Japan) [12]. The total activity was represented by the conversion ratio of LA to *t*10, *c*12-CLA. The relative conversion rate was calculated based on the group with the highest conversion rate as 100%.

$$\mathrm{Conversion}\;\mathrm{Ratio}=\frac{\left[\mathrm{CLA}\right]}{\left[\mathrm{LA}\right]+\left[\mathrm{CLA}\right]}\times 100\%$$

### 2.6. Purification of MBP-PAI and Separation of PAI from the Fusion Tag

Recombinant MBP-PAI insoluble proteins were purified from the crude cell extracts by maltose-affinity chromatography, using an MBPTrap HP (GE Healthcare, Boston, MA. USA). The column was equilibrated with 5 column volumes of binding buffer (100 mmol/L Tris-HCl, 100 mmol/L NaCl, 1 mM EDTA; pH 7.4), and the crude soluble proteins were then pumped into the column. The column was washed with 10 column volumes of binding buffer, and unbound proteins were washed with elution buffer (100 mmol/L Tris-HCl, 100 mM NaCl, 1 mM EDTA, 10 mM maltose; pH 7.4).

The cleavage was carried out at a *w/w* ratio of 5% fusion protein; 0.005~1% SDS or 0.1~5% Factor Xa was added to cleave the fusion protein. The reaction mixtures were incubated for 12 h and 24 h at room temperature, respectively.

### 2.7. Measurement of Purified MBP-PAI Activity

The purified enzyme activity was measured using spectrophotometry (UV-2450; Shimadzu Co., Japan). The enzyme reaction system consisted of 950 μL of buffer F, 60 μg of LA, and 50 μL of purified PAI enzyme solution. The change in absorbance at the 234 nm wavelength over the first minute was determined, and the amount of *t*10, *c*12-CLA was calculated according to the standard product curve at a wavelength of 234 nm. One unit (U) of MBP-PAI activity was defined as the production of 1 nmol CLA within 1 min, and the specific enzyme activity of MBP-PAI was defined as the enzyme activity of 1 mg of purified protein.

### 2.8. Analysis of the Enzymatic Properties of MBP-PAI

The optimum temperature of MBP-PAI was evaluated between 20 °C and 50 °C, and the optimal pH for MBP-PAI activity was assessed from pH 6.5 to pH 9.0. To assess the influence of the metal ions Ca^2+^, Mg^2+^, Co^2+^, Zn^2+^, Ba^2+^, Na^+^, Cu^2+^, Fe^2+^, Fe^3+^, and Ni^+^ on MBP-PAI, the relative enzyme activity of MBP-PAI was determined in buffer F containing 1 mM of metal ions. Four kinds of surfactants were selected to study their effects on enzyme activity, including cationic cetyltrimethylammonium bromide (CTAB), anionic sodium dodecyl sulfonate (SDS), nonionic Tween-20, and Triton X100. The test concentrations of surfactants were 0.1, 0.5, 1, 5, and 10 mM.

The apparent kinetic parameters (*K*m and *V*max) were measured under optimal catalytical conditions, with the tested concentration of LA ranging from 71 μM to 1428 μM. The kinetic parameters *K*m and *V*max were calculated from double reciprocal plots.

## 3. Results and Discussion

### 3.1. Effects of Promoters and Fusion Tags on PAI Expression and Solubility

Strong promoters are the basis for the high-efficiency expression of exogenous genes. However, excessively high transcription rates may cause misfolding of foreign proteins. In order to find a suitable promoter for PAI expression, we investigated three expression systems—pET24a, pColdIV, and pTrc99a—paired with the promoters T7, CspA, and Trc, respectively, representing different transcription strengths. Furthermore, solubility-enhancing tags effectively decreased the proportion of misfolded recombinant proteins in *E. coli* [15]. As a result, the commonly used His6 tag with the pET24a vector, as well as MBP (42 kDa) and Fh8 (8 kDa), were optimized in combination with the promoters. These fusion tags feature solubility and purification properties.

**Figure 3:**
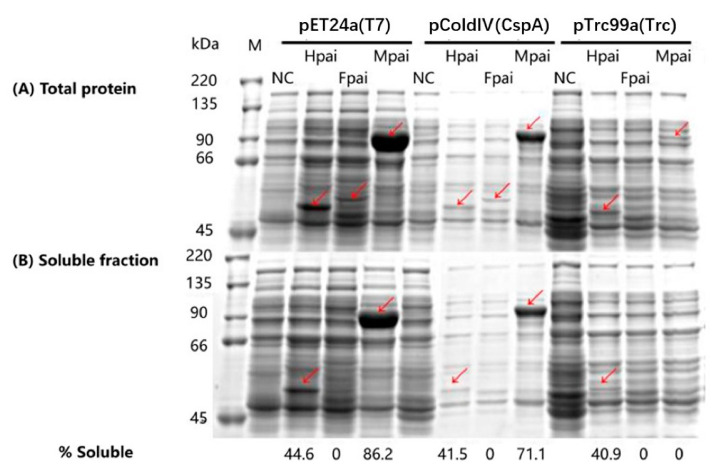
Effects of promoters and fusion tags on total (**A**) and soluble (**B**) expression of PAI, analyzed using SDS-PAGE. Lanes (from left to right): marker, pET24a, T7 + His6, T7 + Fh8, T7 + MBP, pColdIV, CspA + His6, CspA + Fh8, CspA + MBP, pTrc99a, Trc + His6, Trc + Fh8, Trc + MBP. Arrows indicate recombinant PAI protein.

As shown in Figure 3A,B, the highest total expression and soluble expression of recombinant PAI were both achieved in the vector containing the T7 promoter and the MBP fusion tag. When the same fusion tag was used, a higher total recombinant protein expression was observed in the T7 promoter cultures compared to the CspA or Trc promoters (Figure 3A). This result indicated that the total expression level of recombinant protein was closely related to the promoter strength (T7 > CspA > Trc). In contrast, the recombinant PAI solubility did not vary across different promoters (Figure 3B), but significant differences between fusion tags were observed. When His-PAI was paired with the T7, CspA, or Trc promoter, the soluble recombinant protein percentages were 44.6%, 41.5%, and 40.9%, respectively. When MBP-PAI was expressed with T7 or CspA, the soluble recombinant protein percentages were 86.2% and 71.1%, respectively. These results suggested that fusion tags affected the solubility of the fusion protein to a greater extent than the promoters. According to previous research, MBP resulted in the highest solubility, playing a passive role by inhibiting the aggregation of the fusion partners [17]. However, the soluble MBP-PAI could not be detected when paired with the promoter Trc. This may have resulted from the combination of the weaker promoter Trc and larger fusion protein MBP-PAI (92 KD). Moreover, the tag Fh8 was observed to exert a negative effect on PAI folding. Irrespective of the promoter, the soluble Fh8-PAI was not detected by SDS-PAGE. Though Western blot analysis revealed that soluble Fh8-PAI was expressed, its expression levels were significantly lower than those of the fusion His or MBP proteins (data not shown).

### 3.2. Effects of Promoters and Fusion Tags on Total Activity of Crude Enzyme Mixture

In order to study the effects of promoters and fusion tags on the total activity of PAI, the conversion rate of LA into CLA was measured. The complex composition of the cell lysate interferes with the CLA content determination by spectrophotometry. Therefore, gas chromatography was applied for accurate measurement. While measuring the specific activity of the purified PAI, spectrophotometry was used for real-time detection. The strain *E. coli* BL21(DE3) (pET-MBP-PAI) showed the highest conversion rate, and was defined as 100% for comparison with other strains. The relative conversion rate of the crude extract of every recombinant strain was calculated.

**Figure 4:**
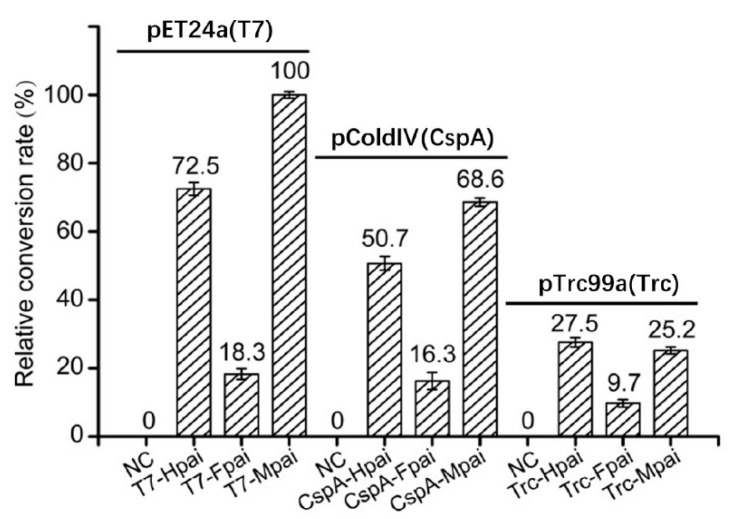
The conversion rate of LA to CLA was catalyzed by different recombinant strains. NC (from left to right): pET24a, pColdIV, pTrc99a. Hpai: His6-PAI, Fpai: Fh8-PAI, Mpai: MBP-PAI.

Consistent with the PAI expression results, the combination of promoter T7 and fusion tag MBP was the most effective in catalyzing LA conversion to CLA. Specifically, in the pET24a system, the conversion rates for MBP-PAI, His-PAI, and Fh8-PAI decreased successively, yielding 100%, 72.5%, and 18.3%, respectively (Figure 4). In the pCold system, the conversion rates for MBP-PAI, His-PAI, and Fh8-PAI showed similar trends, reaching 68.6%, 50.7%, and 16.3%, respectively. In the pTrc99a system, the conversion rates for His-PAI, MBP-PAI, and Fh8-PAI yielded only 27.5%, 25.2%, and 9.7%, respectively. The consistency between the soluble protein expression and the enzyme conversion rate indicated that linoleic acid isomerase activity was retained. The large molecular fusion tag MBP (42 KD) exerted no apparent negative effect on the enzyme activity. In addition, the enzymatic activity results further indicated that all nine recombinant strains expressed active recombinant PAI, including four strains that were undetected by SDS-PAGE.

### 3.3. Purification and Cleavage of MBP-PAI by Factor Xa Protease

**Figure 5:**
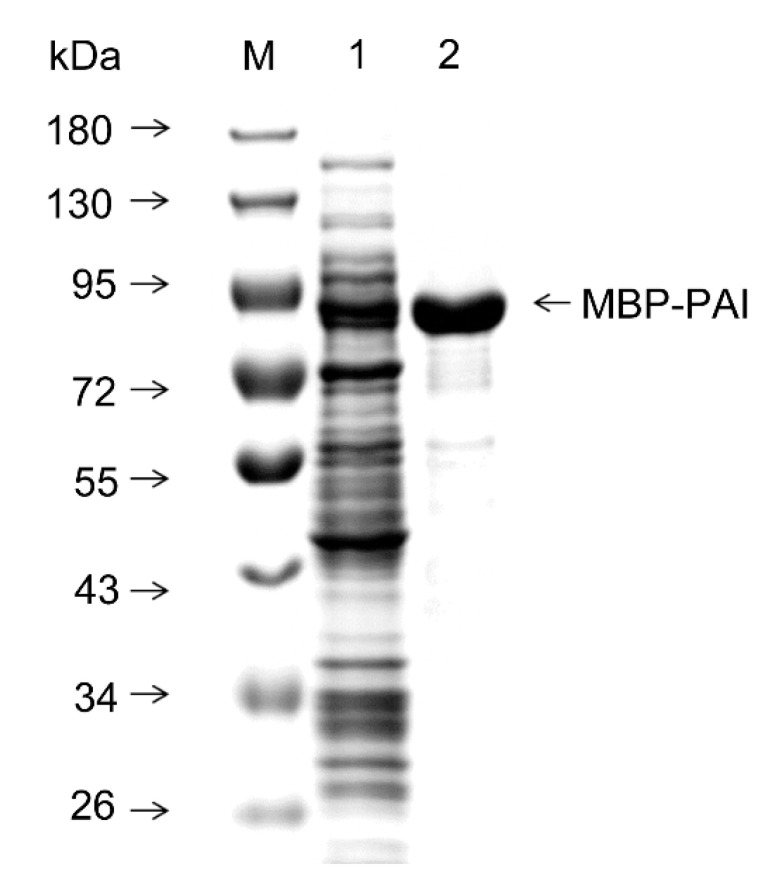
Purification of the expression products of *E. coli* BL21 (DE3) (pET-MBP-PAI). M: marker. Lane 1: crude enzymes after cell lysis; lane 2: purified MBP-PAI.

MBP is a member of the *E. coli* maltose transport system, which can combine maltose and maltodextrin at micromolar levels and can be purified by cross-linked maltose agarose. The soluble MBP-PAI fraction of *E. coli* BL21(DE3) (pET-MBP-PAI) was purified using affinity column chromatography, yielding an MBP-PAI purity exceeding 85% (Figure 5). The resulting concentration of MBP-PAI was 0.46 mg/mL, the enzyme activity yield was 54.5%, and the specific activity was 1084 nmol/min/mg, showing a 41-fold increase after purification (Table 3).

**Figure 6:**
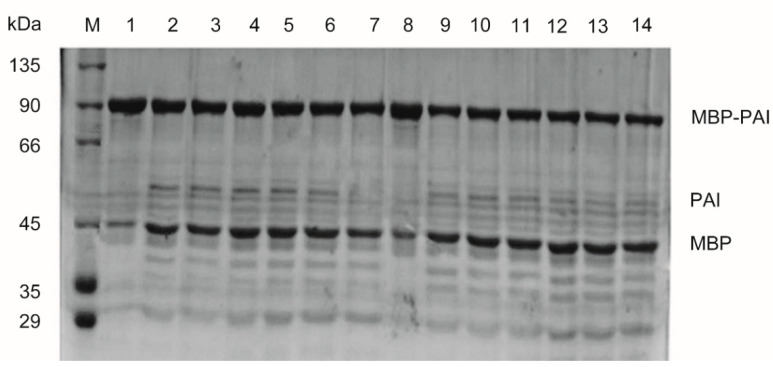
Detection of purified MBP-PAI before cleavage and MBP and PAI after cleavage by Factor Xa. M: marker; Lanes 1 and 8: purified MBP-PAI; lanes 2~7: treated with 1%, 1.5%, 2%, 3%, 4%, and 5% Factor Xa and 0.05% SDS for 12 h; Lanes 9~14: treated with 1%, 1.5%, 2%, 3%, and 4% Factor Xa and 0.05% SDS for 24 h.

The recognition sequence for Factor Xa protease was inserted between the MBP and PAI genes and used to remove the MBP tag from the recombinant fusion protein. The digestion was carried out with different concentrations of Factor Xa protease for 12 h or 24 h. Subsequently, the samples were analyzed with SDS-PAGE. Under all the tested digestion conditions, a new band of 50 kDa (PAI) appeared, while the 40 kDa (MBP) band increased in intensity (Figure 6). However, the 50 kDa band appeared much fainter than the 40 kDa band. Furthermore, multiple bands smaller than 50 kDa were formed following digestion. The results suggested that PAI may be nonspecifically cleaved by Factor Xa protease. The enzyme activity of the recombinant protein was measured to further confirm the findings. After digestion, the total enzyme activity of PAI decreased to 61.2% of that before Factor Xa protease treatment. This also confirmed the nonspecific cleavage by Factor Xa and the reduction in functional PAI. In general, Factor Xa protease specifically recognizes Ile-Glu(Asp)-Gly-Arg sequences, but several studies have indicated that Factor Xa cleavage occurred at additional sites [18,19]. Future research should investigate methods to protect MBP-PAI from nonspecific cleavage by Factor Xa protease.

### 3.4. Characterization of MBP-PAI

**Figure 7:**
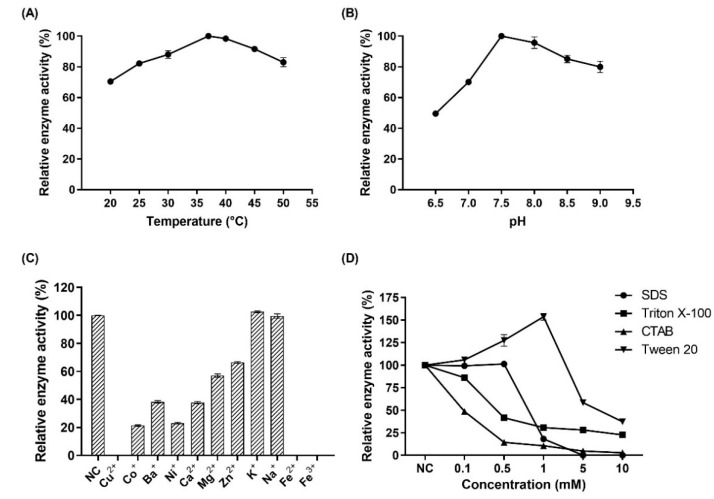
Effect of temperature (**A**), pH (**B**), metal ions (**C**), and surfactants (**D**) on enzyme activity of MBP-PAI.

The characteristics of MBP-PAI were studied comprehensively. The relative activity of the fusion protein MBP-PAI increased with temperatures ranging from 20 °C to 37 °C and decreased at higher temperatures (Figure 7A). The enzyme activity of MBP-PAI is also influenced by pH. The optimum pH for catalyzing MBP-PAI is 7.5, and MBP-PAI maintained high activity (relative activity > 95%) in the pH range of 7.5–8.0 (Figure 7B). The effect of metal ions on the enzyme activity of MBP-PAI was determined in buffer solutions containing 1 mmol/L Ca^2+^, Mg^2+^, Co^2+^, Zn^2+^, Ba^2+^, Na^+^, Cu^2+^, Fe^2+^, Fe^3+^, or Ni^+^. Except for K^+^ and Na^+^, the other eight metal ions decreased the enzyme activity of MBP-PAI. Notably, Cu^2+^, Fe^2+^, and Fe^3+^ caused an almost complete cessation of MBP-PAI catalytic activity (Figure 7C). Due to the hydrophobicity of the substrate LA, surfactants were used to increase the reaction efficiency. Thus, four surfactants were evaluated in the catalytic process. As shown in Figure 7D, Tween-20 (1 mM) increased MBP-PAI activity by 54%. However, when the concentration of Tween-20 was increased to 10 mM, the MBP-PAI activity sharply decreased by 60%. Significant inhibition of enzyme activity was observed with triton X-100, sodium dodecyl sulfate (SDS), and hexadecyl trimethyl ammonium bromide (CTAB). These findings may have been due to competitive inhibition and the denaturing effects of the surfactants on MBP-PAI. Tween 20 exerted positive effects at low concentrations by improving the contact efficiency of the hydrophobic LA with MBP-PAI, achieving a catalytically more favorable conformation of the enzyme [20].

**Figure 8:**
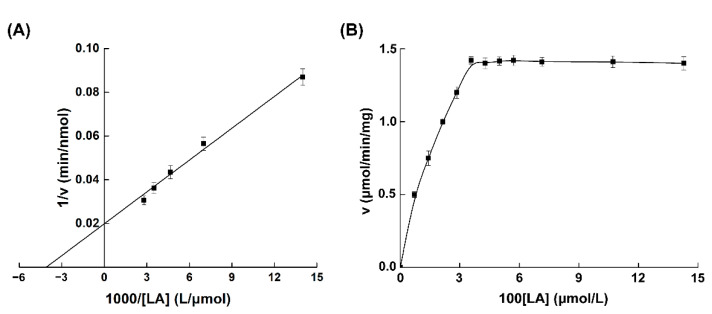
Substrate reaction kinetics of MBP-PAI: (**A**) Lineweaver–Burk plot of MBP-PAI for LA; (**B**) reaction velocity of MBP-PAI at different LA concentrations.

The kinetic parameters of MBP-PAI were determined using the purified enzyme. The regression equation of the Lineweaver–Burk curve was Y = 0.00485X + 0.01989 (R^2^ = 0.9909). The *K*m of MBP-PAI was calculated to be 253.9 μmol/L, and the *V*max was 2253 nmol/min/mg (Figure 8A). In contrast, the *K*m of native PAI from *Propionibacterium acnes* was 17.2 μmol/L [10]. In our previous study, PAI without any tags was heterologously expressed in *Yarrowia lipolityca*, with a *K*m of 84.68 μmol/L (data not published). Compared to the above data, the *K*m of MBP-PAI in this study was higher than those of native PAI and the recombinant PAI without fusion tags. These results indicated that the fusion protein had a low affinity for the substrate. It was speculated that MBP hindered the binding of the substrate and PAI due to its similar molecular weight to PAI. The velocity of MBP-PAI under different concentrations of LA was measured using spectrophotometry. In the first stage, the initial rate increased with an increasing LA concentration. When the substrate concentration was increased from 357μmol/L to 1428 μmol/L, the velocity remained stable (Figure 8B). No substrate inhibition occurred at the substrate concentrations used in this study, which was consistent with the research of Deng et al. [10].

## 4. Conclusions

The low productivity and solubility of PAI from *P. acnes* are the main obstacles limiting its industrial application for the mass production of high value-added *t*10, *c*12-CLA. Although a proper promoter and an effective fusion tag are essential to optimize the expression of target proteins, there is no guaranteed outcome between a given expression system and a particular protein. In this study, different promoters (T7, CspA, Trc) and fusion tags (His, MBP, Fh8) were used to enhance the soluble expression of PAI in *E. coli*. The T7 promoter and MBP tag combination were demonstrated to be the most suitable system out of eight other expression vectors, improving the soluble PAI percentage (86.2%) and obtaining the highest total activity of crude enzyme mixture. The characteristics of MBP-PAI were studied comprehensively. The optimum catalytical conditions of MBP-PAI were 37 °C and pH 7.5 with the addition of 1 mmol/L Tween-20. These results provided an effective strategy to enhance the soluble expression of PAI and promote the industrial production of specific *t*10, *c*12-CLA. It was noticed that we were not able to protect the PAI from degradation during the removal of the MBP tag. In future research, we will improve methods to prevent nonspecific cleavage to realize high-efficiency expression and purification of PAI.

## Figures and Tables

**Table 1:** Primers used for vector cloning. Underlined nucleotides refer to restriction sites or overlaps.

Primer	Sequence	Application Fragment
F1	5′-CGCCATATGCATCATCATCATCATCACATGTCCATCTCGAAGGATTCACG-3′	Fragment *Nde*I-His-PAI- *Xho*I for pET24a(+)
R1	5′-CCGCTCGAGTTACACGAAGAACCGCGTCAC-3′	
F2	5′-CGAGCTCCATCATCATCATCATCACATGTCCATCTCGAAGGATTCACG-3′	Fragment *Sac*I-His-PAI- *Hind*III for pCold IV and pTrc99a
R2	5′-CCCAAGCTTTTACACGAAGAACCGCGTCAC-3′	
F3	5′-CTTTCAAATCAAGCTTATGAAAATCGAAGAAGGTAAAC-3′	Fragment MBP-Factor Xa for pET24a(+)
R3	5′-ACAATTCCCCTCTAGTCACACGAAGAACCGCGTC-3′	
F4	5′-CAAAATTATTTCTAGATGCCTAGTGTTCAAGAGG-3′	Fragment Fh8-Factor Xa for pET24a(+)
R4	5′-CAAGGATGGCAAGCTCCTACCTTCGATCTATGATG-3′	
F5	5′-CCTCGAGGGATCCGAATTCAATGAAAATCGAAGAAGGTAAACTGGTAATC-3′	Fragment MBP-Factor Xa-PAI for pCold IV
R5	5′-TTTAAGCAGAGATTACCTATTCACACGAAGAACCGCGTC-3′	
F6	5′-GCTGGTACCCGGGGATCCATGAAAATCGAAGAAGGTAAACTGGTAATC-3′	Fragment MBP-Factor Xa-PAI for pTrc99a
R6	5′-CTCATCCGCCAAAACAGCCTCACACGAAGAACCGCGTC-3′	
F7	5′-CCTCGAGGGATCCGAATTCATGCCTAGTGTTCAAGAGGTTG-3′	Fragment Fh8-Factor Xa-PAI for pCold IV
R7	5′-TTTAATCATATATTACCTATTCACACGAAGAACCGCGTC-3′	
F8	5′-GCTCGGTACCCGGGGATCCTATGCCTAGTGTTCAAGAGGTTG-3′	Fragment Fh8-Factor Xa-PAI for pTrc99a
R8	5′-CTCATCCGCCAAAACAGAGCCATCACACGAAGAACCGCGTC-3′	

**Table 2:** Induction conditions for the pET-24a(+), pColdIV, and pTrc99a systems.

Expression Vectors	pET24a(+)	pColdIV	pTrc99a
OD_600_	0.6	0.6	0.6
Final concentration of IPTG	0.1 mM	0.1 mM	0.1 mM
Induction temperature	20 °C	15 °C	37 °C
Induction time	20 h	24 h	8 h

**Table 3:** Purification of the MBP-PAI from *E. coli* BL21 (DE3) (pET-MBP-PAI).

**Step**	**Protein** **(mg)**	**Total Activity** **(nmol/min)**	**Specific Activity** **(nmol/min/mg)**	**Yield** **(%)**	**Purification** **(Fold)**
Crude extract	34.8	914	26.3	100	1
Maltose-affinity chromatography	0.461	498	1084	54.5	41

## Data Availability

Data are contained within the article.
